# Potassium-Hydroxide-Based Extraction of Nicotinamide Adenine Dinucleotides from Biological Samples Offers Accurate Assessment of Intracellular Redox Status

**DOI:** 10.3390/ijms262110371

**Published:** 2025-10-24

**Authors:** Tamas Faludi, Daniel Krakko, Jessica Nolan, Robert Hanczko, Akshay Patel, Zach Oaks, Evan Ruggiero, Joshua Lewis, Xiaojing Wang, Ting-Ting Huang, Ibolya Molnar-Perl, Andras Perl

**Affiliations:** 1 Department of Medicine, Norton College of Medicine, State University of New York, Syracuse, NY 13210, USAhanczkor@upstate.edu (R.H.); patel18252@gmail.com (A.P.); ruggieev@upstate.edu (E.R.); wangx1@upstate.edu (X.W.); 2 Department of Microbiology and Immunology, Norton College of Medicine, State University of New York, Syracuse, NY 13210, USA; 3 Department of Biochemistry and Molecular Biology, Norton College of Medicine, State University of New York, Syracuse, NY 13210, USA; 4 Department of Neurology, Stanford University, Palo Alto, CA 94304, USA; 5 Institute of Chemistry, Department of Analytical Chemistry, L. Eötvös University, H-1117 Budapest, Hungary

**Keywords:** NADPH, NADP, NADH, NAD, alkaline extraction, formic acid extraction, methanol extraction, transaldolase, liver, hepatocyte, hepatocellular carcinoma, lupus

## Abstract

The reduced form of nicotinamide adenine dinucleotide phosphate (NADPH) is a primary electron donor for both antioxidant enzymes, such as glutathione reductase, and pro-oxidant enzymes, such as NADPH oxidases that produce reactive oxygen species (ROS) and nitric oxide synthases that generate nitric oxide which act as signaling molecules. Monitoring NADPH levels, NADPH/NADP^+^ ratio, and especially distinguishing from NADH, provides vital information about cellular redox status, energy generation, survival, lineage specification, and death pathway selection. NADPH detection is key to understanding metabolic reprogramming in cancer, aging, and cardiovascular, hormonal, neurodegenerative, and autoimmune diseases. Liquid chromatography combined with mass spectrometry (LC-MS) is crucial for NADPH detection in redox signaling because it offers the high sensitivity, specificity, and comprehensive profiling needed to quantify this vital but labile redox cofactor in complex biological samples. Using hepatoma cell lines, liver tissues, and primary hepatocytes from mice lacking transaldolase or nicotinamide nucleotide transhydrogenase, or having lupus, this study demonstrates that accurate measurement of NADPH depends on its preservation in reduced form which can be optimally achieved by extraction of metabolites in alkaline solution, such as 0.1 M potassium hydroxide (KOH) in comparison to 80% methanol (MeOH) alone or 40:40:20 methanol/acetonitrile/formic acid solution. While KOH extraction coupled with hydrophilic interaction liquid chromatography (HILIC) and mass spectrometry most reliably detects NADPH, NADP, NADH, NAD, polyamines, and polyols, MeOH extraction is best suited for detection of glutathione and overall discrimination between complex metabolite extracts. This study therefore supports performing parallel KOH and MeOH extractions to enable comprehensive metabolomic analysis of redox signaling.

## 1. Introduction

The ability of the cell to combat oxidative stress centers on the production of NADPH, which is indispensable for maintaining a reduced pool of glutathione (GSH), biosynthetic reactions, and maintaining cell membrane integrity [1]. NADPH provides the reducing power for key antioxidant enzymes, such as catalase [2] and glutathione (GSH) reductase [3], which protect cells from oxidative damage [4] throughout evolution from cyanobacteria [5] through mammalian cells [6]. NADPH also acts as essential electron donor for NADPH oxidases (NOX) [7] and nitric oxide (NO) synthases (NOS) that produce critical second messengers in various cellular processes [8], including developmental signaling and immune responses [9]. The balance of NADPH is tightly regulated by production via the pentose phosphate pathway (PPP) [10], malic enzyme [11,12] and isocitrate dehydrogenase [13], and consumption by NADPH-dependent metabolic enzymes, such as NOX, NOS, and aldose reductase [7,14].

The preservation of NADPH/NADP ratio at a high level is paramount in preventing oxidative damage and subsequent cell death during development, tumorigenesis, elimination of virus-infected cells or autoreactive cells [7,15]. NADPH and NADH levels are balanced through transhydrogenation [16]. NADH serves as the major electron donor for the mitochondrial electron transport chain during oxidative phosphorylation [17]. In the liver, the majority of NAD(H) is found in the oxidized form, with NAD/NADH ratios being reported as high as 1000:1 [18], which is considered to drive glycolysis. In other tissues, the ratio of NAD/NADH may be <1 [16,19]. In tumor cells, the absolute quantities and ratios are poorly understood.

The cytoplasmic NADPH/NADP ratio typically varies between 1 and 1000/1, strongly favoring a reducing environment, which is required for the maintenance of cellular integrity [4,18,20]. The accurate measurement of NADPH depends on its preservation in reduced form which has been traditionally achieved by extraction in alkaline solution [14,21,22,23,24,25,26,27]. Recently, extraction with a 40:40:20 acetonitrile:methanol:water solvent with 0.1 M formic acid (FA) has been found to prevent NADPH degradation and minimize interconversion to NADP^+^ during extraction from mammalian cells and mouse liver [28]. As shown in this study, extraction with 0.1 M KOH was found to be optimal relative to that with FA for detection of pyridine nucleotides, NADPH, NADP, NADH, and NAD without detecting reduced glutathione (GSH), another critical antioxidant metabolite. By contrast, MeOH extraction efficiently recovered GSH as well as other polar and semi-polar small molecules, including amino acids, lipids, and glucose metabolites. To maximize metabolome coverage in redox signaling studies, this study advocates for parallel KOH and MeOH extractions, leveraging their complementary chemoselectivity: alkaline KOH extraction efficiently recovers pyridine nucleotides, while MeOH extraction captures a broader spectrum of polar and semi-polar metabolites, including thiols, amino acids, and organic acids. The resulting extracts are analyzed by HILIC–MS, which provides high-resolution separation of polar oxidation-sensitive compounds, thereby enabling comprehensive and quantitative profiling of redox signaling pathways.

## 2. Results

### 2.1. KOH Extraction Is Superior to MeOH Extraction for Detection of Reduced Pyridine Nucleotides

**Figure 1:**
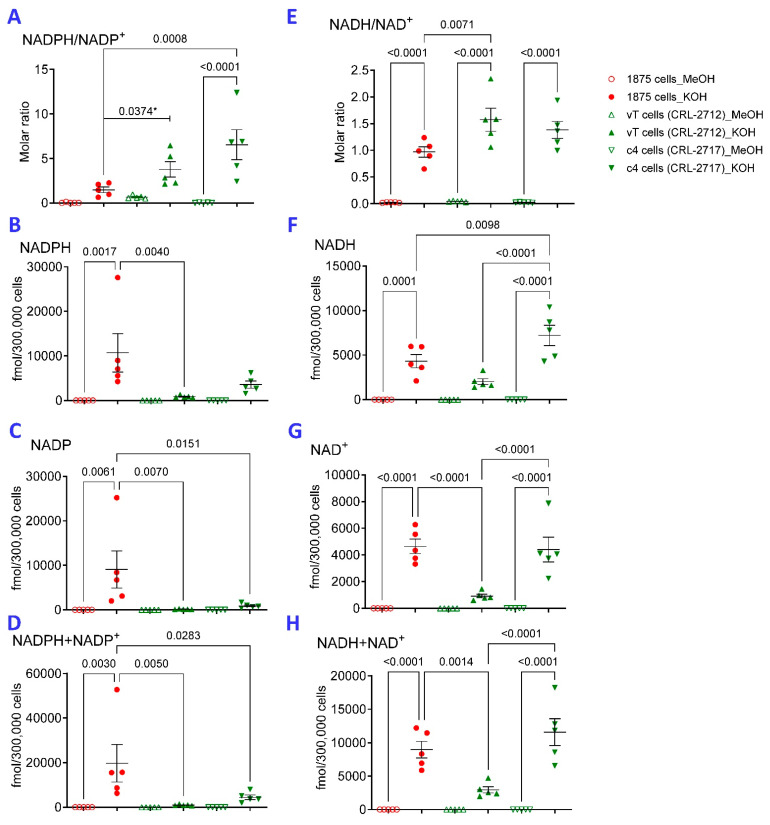
KOH extraction is superior to MeOH extraction for detection of reduced and oxidized pyridine nucleotides in hepatocellular carcinoma (HCC) cell lines. Extraction with 80% methanol (MeOH) and 0.1 M KOH (KOH) were compared for detection of reduced and oxidized pyridine nucleotides in 1875 TALKO, vT, and C4 TAL WT hepatoma cells. NADPH/NADP^+^ (panel (**A**)), NADPH (panel (**B**)), NADP^+^ (panel (**C**)), NADPH, NADP^+^ combined (panel (**D**)), NADH/NAD^+^ (panel (**E**)), NADH (panel (**F**)), NAD^+^ (panel (**G**)), and NADH and NAD^+^ combined (panel (**H**)) were measured by LC-MS. *p* values reflect comparison based on five independent experiments. *, statistical analysis were done with 2-way ANOVA corrected for multiple comparisons with one exception that was done by *t*-test (*).

Extraction with 80% methanol (MeOH) has been widely employed for comprehensive metabolomic studies using LC-MS [29]. To characterize the impact of transaldolase (TAL) [30] on NADPH production by the PPP [10], we compared the influence of methanol extraction to that by 0.1 M KOH which has been used to extract cells and tissues for measurement of pyridine nucleotides by HPLC-UV [25,26,27]. KOH but not MeOH extraction allowed for the detection of diminished NADPH/NADP^+^ in 1875 TALKO over vT and C4 TAL WT hepatomas [31] (Figure 1A). This is consistent with the findings that TAL deficiency blocks the recycling of R5P to G6P for NADPH synthesis via the oxidative branch of the PPP (OxPPPP). Nevertheless, higher NADPH/NADP ratios were detected in each cell line following KOH extraction as compared to MeOH extraction (Figure 1A). Moreover, higher absolute NADPH (Figure 1B) and NADP (Figure 1C) and NADPH + NADP contents were detected following KOH extraction in comparison to MeOH extraction (Figure 1D). Relative to MeOH extraction, KOH extraction also allowed for the detection of higher NADH/NAD ratio (Figure 1E) and greater absolute NADH (Figure 1F), NAD^+^ (Figure 1G), and combined NADH and NAD^+^ content (Figure 1H).

The parental cell line c4 (B13NBii1) (ATCC CRL-2717) lacks functional aryl hydrocarbon receptor nuclear translocator (ARNT) while its vT derivative (ATCC CRL-2712) possesses a complete transfected ARNT cDNA [32]. Notably, NADH (Figure 1F), NAD^+^ (Figure 1G), and combined NADH and NAD^+^ content were all increased in 1875 TALKO and Arnt-deficient c4 HCC cells relative to Arnt-repleted vT cells (Figure 1H).

**Figure 2:**
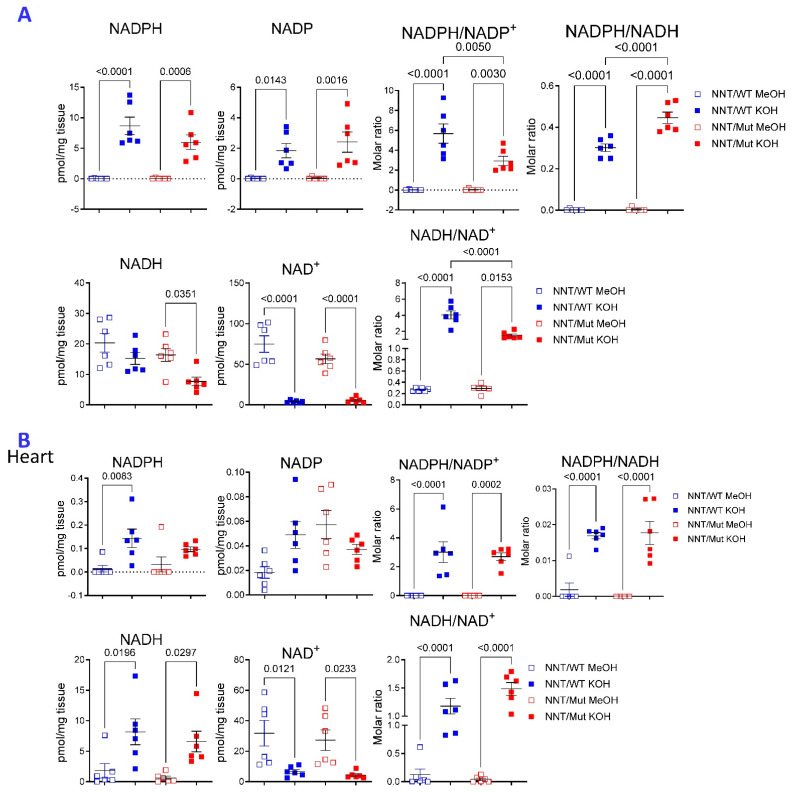
Effect of NNT deficiency on reduced and oxidized pyridine nucleotide content in the liver and heart. KOH and MeOH extractions were performed using liver (panel (**A**)) and heart tissues (panel (**B**)) from six, 10-month-old, age-matched male NNT/WT and NNT/Mut mice. *p* values reflect analysis with one-way ANOVA with Sidak correction for multiple comparisons.

KOH extraction was also superior for detecting NADPH and NADH in liver and heart tissues of commonly used C57Bl/6J mice (B6; Figure 2). Loss of nicotinamide nucleotide transhydrogenase (NNT) in C57BL/6J mice has been attributed to impaired glucose tolerance [33,34], atherosclerosis [35,36], cardiomyopathy [37], and depressive-like behavior [38]. NNT is an integral protein of the inner mitochondrial membrane that catalyzes the transfer of hydrogen between NADPH and NADH [39]. Although the phenotypic differences between the C57BL/6J and parental C57BL/6N strains were initially ascribed to the inactivation of NNT [33], comprehensive analyses identified multiple genomic alterations involving 39 genes [40]. Selective reconstitution of NNT in C57BL/6J mice was found to preserve cardiac function and delay the onset of heart failure under oxidative stress [41]. Other studies found that the absence of NNT alone in C57BL/6J mice afforded protection from oxidative stress, heart failure, and death [42]. Here, we found that NADPH/NADP was increased while NADH/NAD ratios were reduced in the liver of C57Bl/6J mice lacking NNT (NNT/Mut) in comparison to mice carrying functional NNT alleles [41,43] (NNT/WT, Figure 2A). No such differences were noted in the heart (Figure 2B). Moreover, NADPH and NADPH/NADP ratio was also increased in isolated NNT/Mut liver mitochondria (Figure S1). NADH was also increased but the NADPH/NADH ratio remained unchanged in NNT/Mut mitochondria (Figure S1B). MeOH extraction was superior for detection of reduced (GSH) and oxidized glutathione (GSSG) in liver tissues (Figure S2A) and isolated liver mitochondria (Figure S2B). GSH and GSSG were increased but the GSH/GSSG ratio was diminished in NNT/Mut mitochondria (Figure S2B).

### 2.2. Compartmentalized Effect of NNT Deficiency on the Metabolome of the Liver

KOH extraction revealed a compartmentalized impact of NNT within the liver (Figure S3). Besides NADPH and NADP, glycolytic metabolites phosphoenolpyruvate (PEP) and fructose 1,6-bisphosphate (FBP), and TCA metabolites aconitate, citrate, and malate and thiamin phosphate were the main drivers of discrimination between NNT/WT and NNT/Mut mitochondria (Figure S3B–D). Along with reduced GSH/GSSG (Figure S2B), adrenochrome, 8-xox-2-deoxyadenosine, and homocysteine were accumulated in NNT/Mut mitochondria (Figure S3D), suggesting oxidative stress. Depletion of TCA metabolites and PEP and buildup of FBP in NNT/Mut mitochondria suggest that NNT deficiency stimulates glycolysis and elicits a Warburg effect (Figure S3E). Besides major impact on citrate/TCA, pyruvate, and glutathione metabolism, NNT deficiency enhanced tyrosine metabolism (Figure S3F), driven by the accumulation of adrenochrome, and homovanillic acid (Figure S3D). KOH extraction of the liver also allowed for robust discrimination of the metabolomes between NNT/WT and NNT/KO mice (Figure S3G). Adrenochrome was the only metabolite contributing to both VIP scores and biplot separating both mitochondria and whole livers between NNT/WT and NNT/KO mice (Figure S3H–J). Strikingly, NNT deficiency caused the accumulation of adrenochrome in the mitochondria in parallel with its depletion in the liver. Along with adrenochrome, nicotinamide-ribotide, orotate, UDP-xylose, S-adenosyl-methionine (SAM), and 13-OH-α-tocopherol were reduced and dCDP, indol-3-acetate, and taurocholate were accumulated in NNT/Mut livers (Figure S3H–J). NNT deficiency significantly impacted twenty-five biological processes (Figure S3K) and skewed fifteen metabolic pathways at FDR *p* < 0.05 in the liver, with the TCA cycle being most affected (Figure S3L).

MeOH extraction, followed by LC-MS, also effectively distinguished the metabolomes of livers from NNT/WT and NNT/KO mice (Figure S4A). Similarly to KOH extraction, low adrenochrome, homocysteine, and ophthalmic acid indicated reduced oxidative stress in NNT/Mut livers (Figure S4B–D). Reduced argpyrimidine and sedoheptitol also indicated less metabolic stress in NNT deficiency (Figure S4B–D). Testosterone was reduced while progesterone as well as choline phosphate, phosphodimethylethanolamine, cytidine, vitamin D3, and salsolinol-1-carboxylate were accumulated in NNT/Mut livers (Figure S4B–D). Enrichment analysis indicated significant differences in cardiolipin and phospholipid biosynthesis (Figure S4E) while pathway analyses revealed changes in Arg biosynthesis and pyrimidine metabolism (Figure S4F).

### 2.3. The Choice of Extraction Method Impacts the Metabolome Coverage, Which in Turn Affects the Discrimination of Genetically Different NNT/WT and NNT/Mut Mouse Strains

MeOH generally detected greater signal intensities when corrected for recovery of internal standard and tissue mass (Figure S5). However, parallel extractions with MeOH and KOH also allowed for detection of diverse but complementary metabolic abnormalities in NNT deficiency (Figure S5). As revealed by MeOH extraction, loss of NNT reduced metabolism through the mitochondrial tricarboxylic acid (TCA) cycle, glycolysis (Figure S5A), and the pentose phosphate pathway (PPP, Figure S5B). Notably, the accumulation of PPP metabolites, 6-phosphogluconolactone, and C5-polyols, in NNT/Mut livers were detectable after KOH but not MeOH extraction (Figure S5C).

Metabolites involved in pyridine nucleotide biosynthesis, such as kynurenine, kynurenic acid, orotate, inosine, thymine, and xanthosine metabolites were depleted with the exception of IDP, which was accumulated in NNT/Mut livers (Figure S5D). IDP accumulation may be attributed to inhibition of inosine diphosphatase [44].

3-methyladenine was depleted while long chain fatty acid containing carnitines, palmitoyl-carnitine and stearoyl-carnitine, and membrane phospholipids, choline phosphate and phospho-dimethylethanolamine, were accumulated in NNT/Mut livers (Figure S5E).

Loss of NNT caused the depletion of 3-methyladenine, a potent inhibitor of autophagy [45], and the accumulation of long-chain acyl-carnitines, which reflect diminished fatty acid oxidation and mitochondrial dysfunction [46]. The accumulation membrane phospholipids is consistent with enhanced autophagy [47]. While several amino acid metabolites were depleted, salsolinol 1-carboxylate was accumulated in NNT/Mut livers (Figure S5F). Salsolinol 1-carboxylate is a dopamine-derived endogenous alkaloid that can be metabolized into salsolinol, a neurotoxin implicated in Parkinson’s disease [48]. Polyamines, ornithine, putrescine, and spermidine were decreased while spermine was increased in NNT/Mut livers (Figure S5G). Elevated spermine/spermidine ratio is associated with sarcopenia [49], cancer [50], cardiovascular disease, and advanced aging [51]. Quinolinic acid, a kynurenine-derived neurotoxin [52], was also accumulated in NNT/Mut livers (Figure S5H). Prostacyclin was also built up in NNT/Mut livers (Figure S5H), which may have paradoxical anti-inflammatory [53] and neurodegenerative properties [54].

### 2.4. KOH Extraction Is Superior over FA Extraction for Detection of Reduced Pyridine Nucleotides from Hepatocytes

**Figure 3:**
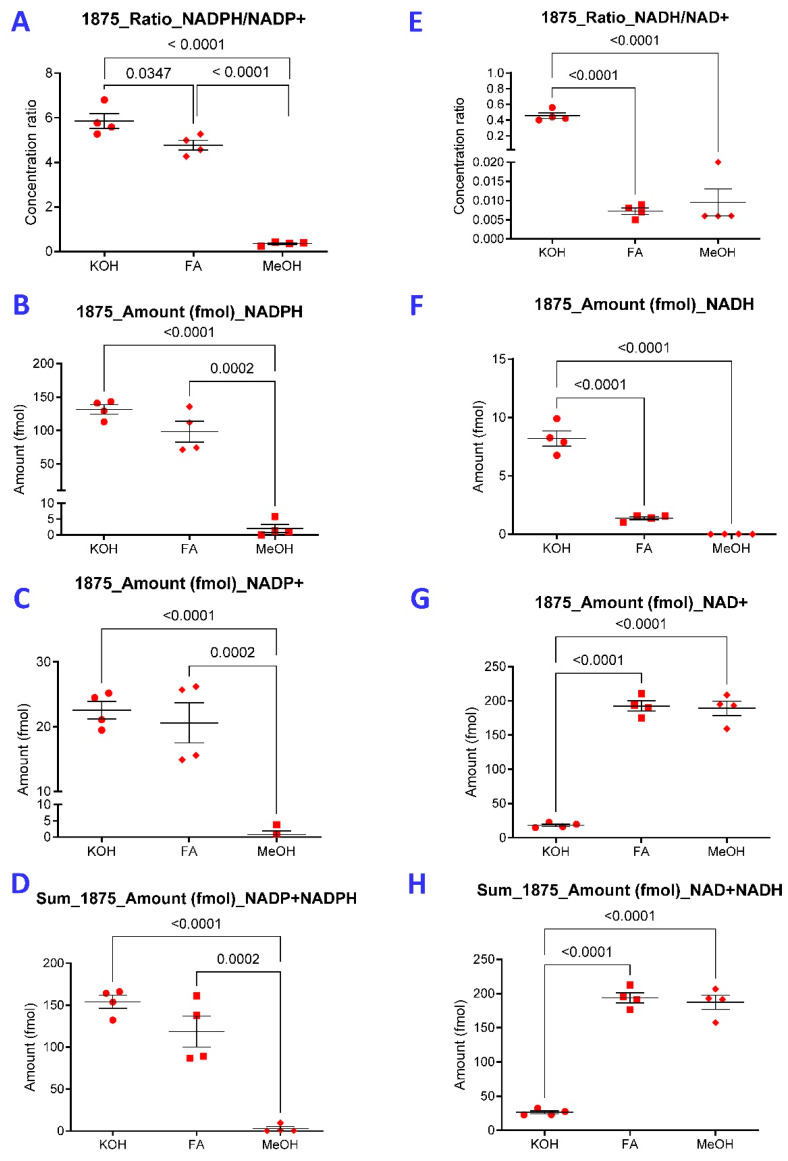
KOH extraction is superior to FA or MeOH extraction for detection of reduced pyridine nucleotides from 1875 TALKO hepatocytes. Extraction with KOH, FA, and MeOH were compared for detection of reduced and oxidized pyridine nucleotides in 1875 TALKO. NADPH/NADP^+^ (panel (**A**)), NADPH (panel (**B**)), NADP^+^ (panel (**C**)), NADPH, NADP^+^ combined (panel (**D**)), NADH/NAD^+^ (panel (**E**)), NADH (panel (**F**)), NAD^+^ (panel (**G**)), and NADH and NAD^+^ combined (panel (**H**)) were measured by LC-MS. *p* values reflect comparison based on five independent experiments.

Next, we compared the performance of NADPH extraction from 1875 TALKO HCC cells with 0.1 M KOH or MeOH to that with 0.1 M formic acid (FA) dissolved in 40:40:20 acetonitrile, methanol, and water [28]. Representative chromatograms of standards and 1875 TALKO HCC cells extracts are shown in Figure S6. FA extraction was found superior to extraction with 80% methanol, hot aqueous solvents, or enzyme assay buffers [28]. As shown in Figure 3A, higher NADPH/NADP molar ratios were detected by KOH over FA extraction, both of which far exceeded those measured by MeOH extraction. NADPH (Figure 3B) and NADP (Figure 3C) and combined NADPH and NADP levels (Figure 3D) were reliably measured via KOH and FA extraction, while they were found profoundly diminished following MeOH extraction (Figure 3A–D). Highest NADH/NAD molar ratios (Figure 3E) and NADH content were also measured following KOH extraction (Figure 3F); however, NAD (Figure 3G) and combined NADH and NAD content were lowest following KOH extraction (Figure 3H).

**Figure 4:**
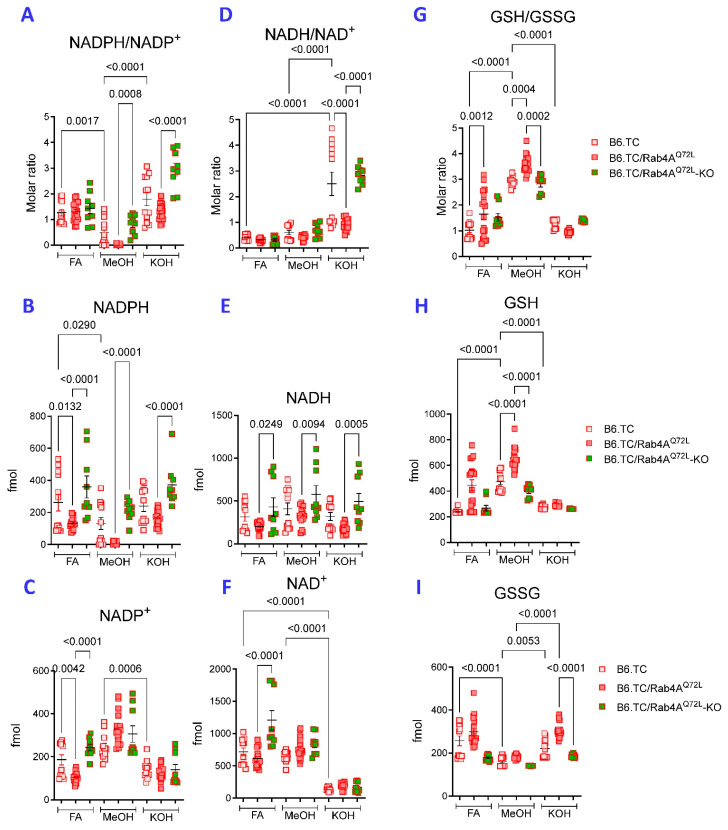
Comparative analyses of KOH, FA, and MeOH extractions for assessing the redox status of pyridine nucleotides and glutathione in hepatocytes from lupus-prone mice. The performance of metabolite extractions was compared in primary hepatocytes isolated from livers of five-month old, age-matched female lupus-prone mice carrying wild-type (B6.TC; n = 12) or constitutively active Rab4A alleles (B6.TC/Rab4A^Q72L^, n = 18) and mice also lacking Rab4A in T cells (B6.TC/Rab4A^Q72L^-KO, n = 9) [55]. NADPH/NADP^+^ (panel (**A**)), NADPH (panel (**B**)), NADP^+^ (panel (**C**)), NADH/NAD^+^ (panel (**D**)), NADH (panel (**E**)), NAD^+^ (panel (**F**)), GSH/GSSG (panel (**G**)), GSH (panel (**H**)), and GSSG (panel (**I**)) were measured by LC-MS. *p* values reflect analysis with one-way ANOVA with Sidak correction for multiple comparisons.

The performance of these three methods of extraction was further compared in primary hepatocytes isolated from livers of lupus-prone mice carrying wild-type (B6.TC) or constitutively active Rab4A alleles (B6.TC/Rab4A^Q72L^) and mice also lacking Rab4A in T cells (B6.TC/Rab4A^Q72L^-KO) [55]. NADPH/NADP^+^ ratios were markedly higher in WT B6.TC mice following FA and KOH extraction relative to MeOH extraction (Figure 4A). Only MeOH and KOH extraction yielded diminished NADPH/NADP^+^ ratio in hepatocytes of B6.TC/Rab4A^Q72L^ mice over B6.TC/Rab4A^Q72L^-KO mice (Figure 4A). However, all methods allowed for the detection of diminished NADPH content in hepatocytes of B6.TC/Rab4A^Q72L^ mice relative to those of B6.TC/Rab4A^Q72L^-KO mice (Figure 4B). MeOH detected less NADPH than FA (Figure 4B) and more NADP^+^ than KOH extraction (Figure 4C). This again suggests that KOH extraction is optimal for detection of NADPH and prevents its degradation to NADP^+^ in primary hepatocytes.

KOH extraction also resulted in markedly increased NADH/NAD^+^ ratio over FA or MeOH extraction in cultured primary hepatocytes (Figure 4D). Moreover, KOH extraction showed decreased NADH/NAD^+^ ratio in B6.TC/Rab4A^Q72L^ hepatocytes relative to B6.TC/Rab4A^Q72L^-KO hepatocytes (Figure 4D). Diminished NADH content of B6.TC/Rab4A^Q72L^ over B6.TC/Rab4A^Q72L^-KO hepatocytes was detected by all three extraction methods (Figure 4E). Notably, KOH extraction detected lower NAD^+^ content relative to FA or MeOH (Figure 4F). While NAD^+^ content was not affected by MeOH or KOH extraction, FA extraction detected elevated NAD^+^ in B6.TC/Rab4A^Q72L^-KO hepatocytes (Figure 4F).

NADPH is essential to maintain glutathione in a reduced state (GSH) [3,10].

Therefore, we also compared GSH, oxidized glutathione (GSSG), and GSH/GSSG ratios, in the same samples used for measurement of pyridine nucleotides. Most glutathione is present in reduced form in hepatocytes at GSH/GSSG ratios of >1:1 to 10:1 [56]. Similarly to liver tissues (Figure S2A), MeOH extraction allowed for the detection of highest GSH/GSSG ratios, while revealing an increased ratio in B6.TC/Rab4A^Q72L^ hepatocytes over B6.TC WT and B6.TC/Rab4A^Q72L^-KO controls (Figure 4G). Along these lines, lower GSH (Figure 4H) and higher GSSG levels were detected after FA and KOH extraction in contrast to MeOH extraction (Figure 4I). These findings suggest that MeOH extraction is optimal in preserving >1 GSH/GSSG ratio in primary hepatocytes.

We also examined the impact of extraction on the numbers of metabolites that were detectable in >75% of samples across all genotypes. KOH extraction detected the largest numbers of distinct compounds across all genotypes (Figure S7A) and allowed for the most distinct separation between genotypes (Figure S7B). The numbers of measurable metabolites were reduced in B6.TC/Rab4A^Q72L^ hepatocytes over B6.TC WT and B6.TC/Rab4A^Q72L^-KO controls (Figure S7B).

### 2.5. NADPH Predominates over NADP in A549 Cells

**Figure 5:**
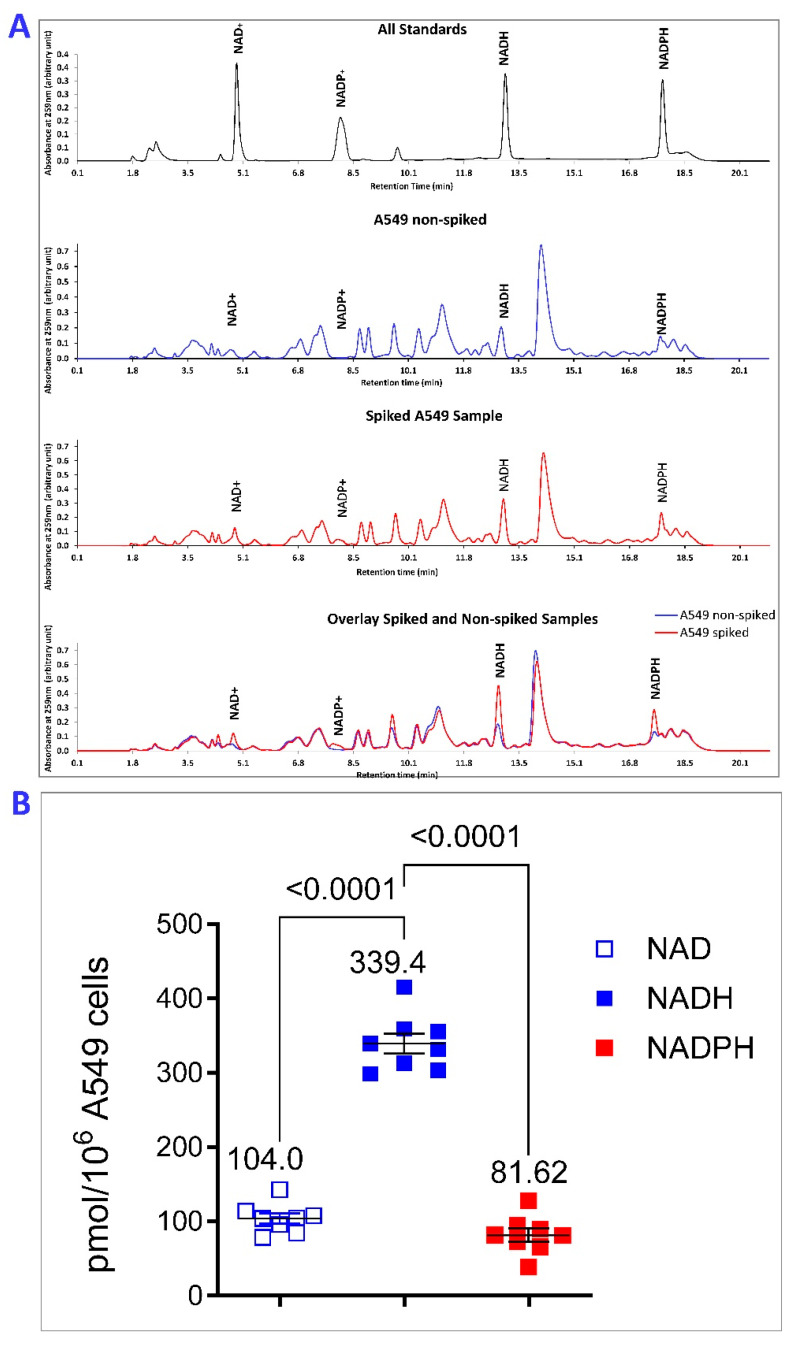
NADPH predominates over NADP in A549 cells. Metabolites were extracted from A549 human lung adenocarcinoma cells with 0.1 M KOH on ice and measured by HPLC using an ultraviolet detector [25]. (**A**), Detection of pyridine nucleotide standards at 259 nm absorbance by HPLC using photodiode array detector. Standard solutions, A549 extracts, and A549 cell extracts spiked with NADPH, NADP^+^, NADH, and NAD^+^ standards were analyzed separately. NADP^+^ was undetectable in 2 × 10^7^ cells. (**B**), Quantitation of NADPH, NADH, and NAD^+^ per 10^6^ A549 cells. Results and *p* values reflect 8 independent experiments.

Baseline NADP^+^/NADPH ratios of 7–14 [57] and 2:1 were measured by enzymatic recycling assays in A549 human lung adenocarcinoma cells when metabolites were extracted at 60 °C [57] or 4 °C, respectively [58]. Alternatively, NADPH but not NADP was detectable in A549 cells when cells were extracted on ice and measured by an enzymatic recycling method [59]. Following KOH extraction on ice [26], we detected all pyridine nucleotide standard solutions at 259 nm absorbance using HPLC-UV (Figure 5A). With this extraction method, we found 81.6 ± 10 pmol of NADPH per million A549 cells whereas the concentration of NADP^+^ was below our limit of detection (Figure 5B). We could detect a minimum of 20 pmol of NADP^+^; therefore, the ratio of NADPH/NADP^+^ was not less than 4:1, i.e., the NADP^+^/NADPH is ≤0.25. This indicates that NADPH still predominates over NADP^+^ in A549 cells. At 259 nm absorbance, we detected 104.0 ± 5 pmol of NAD^+^ and 339.4 ± 17 pmol of NADH per million A549 cells, resulting in a NAD^+^/NADH ratio of 0.31 (Figure 5B).

## 3. Discussion

The cytoplasmic NADPH/NADP^+^ ratio typically varies between 1 and 1000/1, strongly favoring a reducing environment, which is required for the maintenance of cellular integrity [4,18,20,60,61]. Reliable measurement of NADPH depends on its preservation in reduced form which has long been achieved by extraction in alkaline solution [14,21,22,23,24,25,26,27,62]. Alternatively, extraction with 80% methanol (MeOH) has been widely employed for comprehensive metabolomic studies using LC-MS [29,63,64,65,66]. Methanol extraction has been found to yield the largest numbers of reliably detectable metabolites in mammalian cells and tissues [29,66]. However, we typically failed to detect NADPH in MeOH extracts and separately employed KOH extraction to detect pyridine nucleotides by enzymatic recycling [24], HPLC-UV [25], or LC-MS [14,26,27,55]. The present results confirm that the choice of metabolite extraction may lead to common and substantial mismeasurements of absolute NADPH content and NADPH/NADP^+^ ratios [64].

The method of extraction did not appear to have a major effect on the total numbers of metabolites; however, significant qualitative differences have been detected. While KOH extraction was found to be optimal for measurement of pyridine nucleotides, MeOH extraction was better suited for detecting GSH and >1 GSH/GSSG ratios, which was reflective of the reducing intracellular environment. GSH/GSSG ratio was elevated in B6.TC/Rab4A^Q72L^ hepatocytes over B6.TC controls following FA and MeOH but KOH extraction. These findings suggest that KOH may not be suitable for detection of GSH and GSSG. Although not addressed in this study, derivatization of GSH sulfhydryl groups is required to allow detection of GSH after long-term storage [67].

Relative to KOH, MeOH extraction requires the least number of experimental steps. MeOH also resulted in greater signal intensities allowing for the detection of greater numbers of discordant metabolites in the liver. Moreover, KOH extraction involves neutralization with KHCO_3_, which results in the formation of K_2_CO_3_ precipitate. This requires an extra step of filtration and more frequent flushing of the tubing system and column replacement.

Loss of NNT in C57BL/6J mice has been attributed to impaired glucose tolerance [33,34], atherosclerosis [35,36], cardiomyopathy [37], depressive-like behavior [38], and impaired steroidogenesis in male mice [68]. However, these studies, associating loss of NNT with disease pathogenesis, compared the C57BL/6J mouse strain with C57BL/6N without adequate control for other genetic differences [40]. Although the phenotypic differences between the C57BL/6J and C57BL/6N strains were initially ascribed the inactivation of NNT [33], comprehensive analyses identified multiple genomic alterations involving 39 genes [40]. Similarly to our findings in this study, increased NADPH/NADP^+^ ratio was attributed to low NADP^+^ content in liver mitochondria of C57BL/6J mice relative to C57BL/6N controls [13]. Moreover, mitochondrial GSH/GSSG was reduced in NNT mutant mitochondria of C57BL/6J mice relative to C57BL/6N controls [13]. These findings suggest that the increased NADPH/NADP^+^ ratio may paradoxically elicit oxidative stress in liver mitochondria in the absence of NNT.

Selective reconstitution of NNT in C57BL/6J mice was found to preserve cardiac function and delay the onset of heart failure under oxidative stress [41]. Other studies found that the absence of NNT alone in C57BL/6J afforded protection from oxidative stress, heart failure, and death [42]. NNT was found to transfer hydrogen from NADPH to NADH in uncoupled mitochondria and reduce GSH/GSSG ratio and increase oxidative stress in the overloaded heart [42].

This study suggests that metabolomics studies aimed at addressing the role of NADPH in redox signaling should rely on several independent methods of extraction. We repeatedly found that KOH extraction is optimal to preserve NADPH in its reduced state [14,24,25,26,27]. Given that NADPH degrades at higher temperatures (>50 °C) [64,69], incubation of cell extracts at 60 °C for 30 min is likely to have caused the spurious detection of baseline NADP^+^/NADPH ratios of 7–14 in A549 cells [57]. Phenol-chloroform-isoamyl alcohol extraction has been successfully used for quantifying NADPH/NADP^+^ in cyanobacteria [70]. However, this method involves using hazardous, volatile chemicals, and sample processing in a chemical hood. The method of detection by enzymatic recycling [24,42], HPLC with UV detection [25,26], or LC-MS are equally suitable for reliable, high-throughput analysis [14].

With respect to medical relevance, HCC is the fifth most common cancer and the third leading cause of cancer-related mortality worldwide [71]. Liver cancer, which included HCC, accounted for 0.74 million cases, 0.48 million deaths, and 12.89 million disability-adjusted life years (DALYs) globally [72]. High-income North America and Western Europe experienced rapid growth in liver cancer prevalence from 1990 to 2021, while high-income North America and Southern Latin America had rapid growth in mortality. Global DALY increases were mainly driven by population growth (3.91 million, 73.29%) and population aging (3.03 million, 56.86%). Systemic lupus erythematosus (SLE) affects 0.1% of the US population, mainly women of child-bearing age, with 10% mortality in 5 to 10 years [73]. While a single, recent global DALY figure for SLE is not readily available, recent data from a 2025 Global Burden of Disease study analysis on musculoskeletal disorders places SLE burden (Years Lived with Disability, YLDs) at 40.7 million (95% UI: 34.1–45.7 million) globally, representing a significant portion of total musculoskeletal DALYs. This reflects the disease’s significant impact on quality of life, with patients often experiencing severe fatigue and physical pain that limits daily activities [74,75].

## 4. Materials and Methods

### 4.1. Metabolite Extraction from Plate Bound Hepatocytes

#### 4.1.1. Methanol Extraction

The 6-well plates containing the adherent cells were placed on ice, the media was aspirated from the wells, and the cell monolayers were washed with 1 mL ice-cold PBS solution (pH 7.4). Then, plates were placed on dry ice, 400 µL 80:20 (*v*/*v*) methanol:water (precooled at −80 °C) was added to the wells, and cells were scraped with a disposable cell scraper (ThermoFisher Scientific, Pittsburgh, PA, USA; Cat# 08-100-242) to detach them from the well surface. The extraction solvent contained 4-thio-glucose as internal standard in 2.25 µM concentration. After scraping, the plates were kept on dry ice for an additional 5 min, then cell extracts were transferred into 1.5 mL microcentrifuge tubes (ThermoFisher Scientific Cat# 05-402-94), placed to −80 °C for 15 min, after which they were centrifuged at 13,000 rpm for 30 min at 4 °C. In the next step, the supernatants were transferred to new tubes, evaporated to dryness using a SpeedVac vacuum concentrator (Savant AS160, Farmingdale, NY, USA), and stored at −80 °C. On the day of analysis, samples were redissolved in 75 µL ice-cold methanol, briefly vortexed, and filtered directly into fused insert autosampler vials (ThermoFisher Scientific Cat# C4000-LV2W) through 0.20 µm pore size Millex PTFE membrane syringe filters (Sigma-Aldrich, Saint Louis, MO, USA; Cat# SLFGR04NL). The vials were placed into the cooled autosampler of the liquid chromatograph at 4 °C.

#### 4.1.2. FA Extraction

The 6-well plates containing the adherent cells were placed on ice, the media was aspirated from the wells, and the cell monolayers were washed with 1 mL ice-cold PBS solution. Then, 400 uL of ice-cold solution of acetonitrile, MeOH, and 0.1M formic acid (FA) at 40/40/20 ratio with IS was added directly to the plates. Cell scraper was used to thoroughly detach the cells. After scraping, the cell extracts were transferred into 1.5 mL microcentrifuge tubes, subjected to two freeze (−80 °C for 15 min) and thaw cycles, neutralized with 35 µL 15% ice-cold ammonium (NH_4_)HCO_3_ solution, vortexed, then centrifuged at 13,000 rpm for 30 min at 4 °C. After centrifugation, the supernatants were transferred to new tubes, evaporated to dryness in a SpeedVac vacuum concentrator, and stored at −80 °C. On the day of analysis, samples were redissolved in 125 µL ice-cold methanol, vortexed, and filtered directly into fused insert autosampler vials through 0.20 µm pore size Millex PTFE membrane syringe filters. The vials were placed into the cooled autosampler of the liquid chromatograph at 4 °C.

#### 4.1.3. KOH Extraction

The 6-well plates containing the adherent cells were placed on ice, the media was aspirated from the wells, and the cell monolayers were washed with 1 mL ice-cold PBS solution. Then, 400 µL 0.5 M ice-cold KOH solution and 20 µL 4-thio-glucose solution (internal standard) were added to the wells and cells were scraped with a disposable cell scraper (ThermoFisher Scientific Cat# 08-100-242) to detach them from the well surface. After scraping, the cell extracts were transferred into 1.5 mL microcentrifuge tubes, subjected to two freeze (−80 °C for 15 min) and thaw cycles, neutralized with 80 µL 2.5 M ice-cold KHCO_3_ solution, vortexed, then centrifuged at 13,000 rpm for 30 min at 4 °C. After centrifugation, the supernatants were transferred to new tubes, evaporated to dryness in a SpeedVac vacuum concentrator and stored at −80 °C. On the day of analysis, samples were redissolved in 125 µL ice-cold methanol, vortexed, and filtered directly into fused insert autosampler vials through 0.20 µm pore size Millex PTFE membrane syringe filters. The vials were placed into the cooled autosampler of the liquid chromatograph at 4 °C.

The KOH extraction methods were optimized in our laboratory over several decades for detection of NADPH by enzymatic recycling [24], HPLC [25,27], and LC-MS [14,26]. We experimented with 0.3 M KOH [27,76] and 0.5 M KOH [14]. Others also used KOH concentrations higher than 0.1 M, such as 0.5 M [77,78].

### 4.2. Metabolite Extraction from Suspension Cells

#### 4.2.1. Methanol Extraction

Cell suspensions were transferred into 1.5 mL microcentrifuge tubes (ThermoFisher Scientific Cat# 05-402-94), centrifuged at 500 rcf for 5 min at 4 °C, and the supernatants were aspirated. Then, 400 µL 80:20 (*v*/*v*) methanol:water (precooled at −80 °C), containing 2.25 µM 4-thio-glucose solution (internal standard), was added to the pellets. In the next step, samples were briefly vortexed, placed to −80 °C for 15 min, then centrifuged at 13,000 rpm for 30 min at 4 °C. After centrifugation, the supernatants were transferred into new tubes, evaporated to dryness using a SpeedVac vacuum concentrator (Savant AS160, Farmingdale, NY, USA), and stored at −80 °C. On the day of analysis, samples were redissolved in 75 µL ice-cold methanol, briefly vortexed, and filtered directly into fused insert autosampler vials (ThermoFisher Scientific Cat# C4000-LV2W) through 0.20 µm pore size Millex PTFE membrane syringe filters (Sigma-Aldrich Cat# SLFGR04NL). The vials were placed into the cooled autosampler of the liquid chromatograph at 4 °C.

#### 4.2.2. KOH Extraction

Cell suspensions were transferred into 1.5 mL microcentrifuge tubes (ThermoFisher Scientific Cat# 05-402-94), centrifuged at 500 rcf for 5 min at 4 °C, and the supernatants were aspirated. Then, 400 µL 0.5 M ice-cold KOH solution and 20 µL 4-thio-glucose solution (internal standard) were added to the pellets. In the next step, samples were briefly vortexed and subjected to two freeze (−80 °C for 15 min) and thaw cycles, neutralized with 80 µL 2.5 M ice-cold KHCO_3_ solution, vortexed, then centrifuged at 13,000 rpm for 30 min at 4 °C. After centrifugation, the supernatants were transferred into new tubes, evaporated to dryness using a SpeedVac vacuum concentrator (Savant AS160, Farmingdale, NY, USA), and stored at −80 °C. On the day of analysis, samples were redissolved in 125 µL ice-cold methanol, briefly vortexed, and filtered directly into fused insert autosampler vials (ThermoFisher Scientific Cat# C4000-LV2W) through 0.20 µm pore size Millex PTFE membrane syringe filters (Sigma-Aldrich Cat# SLFGR04NL). The vials were placed into the cooled autosampler of the liquid chromatograph at 4 °C.

### 4.3. Metabolite Extraction from Mitochondria

#### 4.3.1. Isolation of Mitochondria

Liver mitochondria were isolated by differential centrifugation as previously described [79]. Livers were removed, cut into small pieces which were mechanically disrupted in a 15 mL Dounce homogenizer on ice in liver mitochondria isolation medium (LMIM) containing 250 mM sucrose, 10 mM Tris, 1 mM EGTA, pH 7.4. Homogenate was centrifuged at 1000× *g* for 3 min at 4 °C. The supernatant was transferred to new tubes and then centrifuged at 10,000× *g* for 10 min at 4 °C. The pellet was resuspended in LMIM and centrifuged again at 10,000× *g* for 10 min at 4 °C. The mitochondrial pellet was then resuspended in a Ca^2+^ chelating buffer containing 195 mM mannitol, 25 mM sucrose, 40 mM HEPES pH 7.2, 1 mM EGTA, 10 mM NaCl, and 5 mM succinate at room temperature. This mitochondrial solution was homogenized in a 2 mL Dounce homogenizer and then stirred at room temperature for 10 min and then on ice for 5 min. Mitochondria were centrifuged at 10,000× *g* for 10 min at 4 °C and resuspended in liver swelling buffer (LSB), which contained 195 mM mannitol, 25 mM sucrose, 40 mM HEPES pH 7.2 at 4 °C. Mitochondria were centrifuged at 10,000× *g* for 10 min at 4 °C twice and then resuspended in 1 mL of LSB and protein content was measured by Bradford assay [80]. Mitochondria were kept on ice for 1 h before using in downstream analyses. NaCl, 4-(2-hydroxyethyl)-1-piperazineethanesulfonic acid (HEPES), sucrose, and ethylene glycol-bis(2-aminoethylether)-N,N,N′,N′-tetraacetic acid (EGTA) were obtained from Sigma-Aldrich (St. Louis, MO, USA). Tris-HCl was obtained from USB (Cleveland, OH, USA). Succinic acid was obtained from Acros Organics (Geel, Belgium). Isolation of mitochondria was confirmed by Western blot detection of proteins involved in oxidative phosphorylation (Oxphos antibody coctail, Abcam, Waltham, MA, USA; Cat. No. 110413). Purity of mitochondrial fraction from cytosol was assessed by Western blot detection of cytosolic enzyme, transaldolase (TAL), using rabbit polyclonal antibody 169 [26]. NNT was detected with a rabbit polyclonal antibody (Sigma Cat. No. HPA004829).

#### 4.3.2. Methanol Extraction

Mitochondria (400 µL) were aliquoted into 2 mL microcentrifuge tubes (Krackeler, Albany, NY, USA; Cat# 383-MCT200C), placed on ice and 1000 µL 80:20 (*v*/*v*) methanol:water (precooled at −80 °C), containing 2.25 µM 4-thio-glucose solution (internal standard), was added to them. In the next step, samples were briefly vortexed, placed to −80 °C for 15 min, then centrifuged at 13,000 rpm for 30 min at 4 °C. After centrifugation, the supernatants were transferred into new tubes, evaporated to dryness using a SpeedVac vacuum concentrator (Savant AS160, Farmingdale, NY, USA), and stored at −80 °C. On the day of analysis, samples were redissolved in 75 µL ice-cold methanol, briefly vortexed, and filtered directly into fused insert autosampler vials (ThermoFisher Scientific Cat# C4000-LV2W) through 0.20 µm pore size Millex PTFE membrane syringe filters (Sigma-Aldrich Cat# SLFGR04NL). The vials were placed into the cooled autosampler of the liquid chromatograph at 4 °C.

#### 4.3.3. KOH Extraction

Mitochondria (400 µL) were aliquoted into 2 mL microcentrifuge tubes (Krackeler Cat# 383-MCT200C), placed on ice and 1000 µL 0.5 M ice-cold KOH solution, and 20 µL 4-thio-glucose solution (internal standard) was added to them. In the next step, samples were briefly vortexed, and subjected to two freeze (−80 °C for 15 min) and thaw cycles, neutralized with 80 µL 2.5 M ice-cold KHCO_3_ solution, vortexed, then centrifuged at 13,000 rpm for 30 min at 4 °C. After centrifugation, the supernatants were transferred into new tubes, evaporated to dryness using a SpeedVac vacuum concentrator (Savant AS160, Farmingdale, NY, USA), and stored at −80 °C. On the day of analysis, samples were redissolved in 125 µL ice-cold methanol, briefly vortexed, and filtered directly into fused insert autosampler vials (ThermoFisher Scientific Cat# C4000-LV2W) through 0.20 µm pore size Millex PTFE membrane syringe filters (Sigma-Aldrich Cat# SLFGR04NL). The vials were placed into the cooled autosampler of the liquid chromatograph at 4 °C.

### 4.4. Metabolite Extraction from Organ Tissue

#### 4.4.1. Methanol Extraction

Approximately 50–100 mg frozen mouse tissue (liver, brain, or heart) was weighed and placed into 2 mL microcentrifuge tubes (Krackeler Cat# 383-MCT200C); the tubes were placed on dry ice. Then, 400 µL 80:20 (*v*/*v*) methanol:water (precooled at −80 °C), containing 2.25 µM 4-thio-glucose solution (internal standard), was added; samples were vortexed, then homogenized using a Power Gen 500 (Fisher Scientific, Waltham, MA, USA). In the next step, samples were placed to −80 °C for 15 min, then centrifuged at 13,000 rpm for 30 min at 4 °C. After centrifugation, the supernatants were transferred into new tubes, evaporated to dryness using a SpeedVac vacuum concentrator (Savant AS160, Farmingdale, NY, USA), and stored at −80 °C. On the day of analysis, samples were redissolved in 75 µL ice-cold methanol, briefly vortexed, and filtered directly into fused insert autosampler vials (ThermoFisher Scientific Cat# C4000-LV2W) through 0.20 µm pore size Millex PTFE membrane syringe filters (Sigma-Aldrich Cat# SLFGR04NL). The vials were placed into the cooled autosampler of the liquid chromatograph at 4 °C.

#### 4.4.2. KOH Extraction

Approximately 50–100 mg frozen mouse tissue (liver, brain, or heart) was weighed and placed into 2 mL microcentrifuge tubes (Krackeler Cat# 383-MCT200C); the tubes were placed on dry ice. Then, 400 µL 0.5 M ice-cold KOH solution and 20 µL 4-thio-glucose solution (internal standard) were added, samples were vortexed, then homogenized using a Power Gen 500 (Fisher Scientific, Waltham, MA, USA). In the next step, samples were subjected to two freeze (−80 °C for 15 min) and thaw cycles, neutralized with 80 µL 2.5 M ice-cold KHCO_3_ solution, vortexed, then centrifuged at 13,000 rpm for 30 min at 4 °C. After centrifugation, the supernatants were transferred into new tubes, evaporated to dryness using a SpeedVac vacuum concentrator (Savant AS160, Farmingdale, NY, USA), and stored at −80 °C. On the day of analysis, samples were redissolved in 125 µL ice-cold methanol, briefly vortexed, and filtered directly into fused insert autosampler vials (ThermoFisher Scientific Cat# C4000-LV2W) through 0.20 µm pore size Millex PTFE membrane syringe filters (Sigma-Aldrich Cat# SLFGR04NL). The vials were placed into the cooled autosampler of the liquid chromatograph at 4 °C.

### 4.5. High-Performance Liquid Chromatography with Ultraviolet Detection (HPLC-UV)

A549 cells were obtained from ATCC and were cultured adherently. 2 × 10^7^ cells were aliquoted into individual tubes, washed with PBS, pelleted, and resuspended in 250 µL of 0.5 M ice-cold KOH. The cells were then freeze-thawed three times. Lysates were then buffered using 1/5 volume of 2.5 M KHCO_3_ and pelleted. Lysates were then filtered through a 0.45 µm PVDF filter. A total 80 µL of lysates were injected using a Waters 2695 HPLC (Milford, MA, USA) equipped with a Model 996 photodiode array detector. We acquired ultraviolet (UV) signal at 259 nm to detect all pyridine nucleotides and at 338 nm to detect reduced pyridine nucleotides.

### 4.6. High-Performance Liquid Chromatography—Mass Spectroscopy (LC-MS)

LC-MS measurements were performed on a Thermo Scientific Vanquish HPLC coupled to a Thermo Scientific Q Exactive hybrid quadrupole-orbitrap MS. The metabolites were separated using a hydrophilic interaction liquid-chromatography (HILIC) method on a Waters Xbridge BEH Amide column (3.5 µm, 2.1 × 100 mm, P/N: 186004860; Milford, MA, USA) kept at 30 °C during the analysis. Mobile phase component A was 10 mM ammonium acetate and 7.5 mM ammonium hydroxide in water with 3% (*v*/*v*) acetonitrile (pH 9.0) while mobile phase component B was 100% acetonitrile. The 25 min-long gradient was as follows: 0 min, 85% B; 1.5 min, 85% B; 5.5 min, 35% B; 14.5 min, 35% B; 15.0 min, 85% B; 25.0 min, 85% B. The mobile phase flow rate was the following: 0 min, 0.150 mL/min; 10.0 min, 0.150 mL/min; 10.5 min, 0.300 mL/min; 14.5 min, 0.300 mL/min; 15.0 min, 0.150 mL/min, 25.0 min, 0.150 mL/min. The sample injection volume was 5 µL. The Thermo Scientific Q Exactive MS was operated in polarity switching mode throughout the acquisition run to maximize metabolite coverage. The heated-electrospray ionization (HESI) probe parameters were the following in both polarity modes: sheath gas flow rate 30, aux gas flow rate 10, sweep gas flow rate 0, spray voltage 3.60 kV, aux gas heater temp 120 °C, S-lens RF level 55, ion transfer capillary temp 320 °C. In positive mode, the instrument acquired Full Scan spectra with a *m*/*z* range of 61–915, while in negative mode, the Full Scan mass range was *m*/*z* 70–920. The resolution of the scans was 70,000, the AGC target was 3 × 10^6^, while maximum injection time was 200 ms. Based on the processing of NADPH standards alone or tissue extracts spiked with NADPH standards, neither the recovery nor the purity of the metabolite was affected by extraction with 0.1 M KOH.

### 4.7. Metabolite Steady-State, Pathway, and Statistical Analyses

Quantitative enrichment of detected metabolites was utilized for pathway analysis employing the web-based MetaboAnalyst 6.0 software [81]. Samples from compared mice were matched for age and gender and were injected in the same LC-MS/MS run. The signal stability was assured by normalizing the controls between runs to the sum of all signals between separate runs using MetaboAnalyst. The enrichment analysis was based on global analysis of covariance (Ancova). A Google map style interactive visualization system was utilized for data exploration and creation of a three-level graphical output: metabolome view, pathway view, and compound view. The ‘metabolome view’ shows all metabolic pathways arranged according to the scores from enrichment analysis (y axis: −log *p*) and from topology analysis (x axis: impact: number of detected metabolites with significant *p* value). The pathway topology analysis used two well-established node centrality measures to estimate node importance: degree centrality and betweenness centrality. Degree centrality depends on the number of links connected to a given node. For directed pathway graphs, there are two types of degrees: in-degree for links that came from other nodes, and out-degree for links initiated from the current node. Here, we only considered the out-degree for node importance measure. Upstream nodes are considered to have regulatory roles for the downstream nodes, and not vice versa. The betweenness centrality measures the number of shortest paths going through the node. Since metabolic networks are directed, we used relative-betweenness centrality for a metabolite importance measure based on metabolite topology weighed by relative-betweenness centrality [81]. The degree centrality measures focus more on local connectivity, while the betweenness centrality measures focus more on global network topology. The node importance values calculated from centrality measures were further normalized by the sum of the importance of the pathway. Therefore, the total/maximum importance of each pathway reflects the importance measure of each metabolite node that is actually the percentage relative to the total pathway importance, and the pathway impact value is the cumulative percentage from the matched metabolite nodes. The altered compounds have been grouped and presented together for each pathway.

Metabolite concentrations were evaluated for their ability to discriminate between WT, Rab4A-KI, and Rab4A-KO mice by partial least squares-discriminant analysis (PLS-DA) [82]. Contribution of individual metabolites to PLS-DA was assessed by variable importance in projection (VIP) and coefficient scores. Individual compounds were also compared between B6 and lupus-prone mice by two-way ANOVA paired and Tukey’s correction for multiple comparisons using Prism Software Version 10 (GraphPad, San Diego, CA, USA).

## 5. Conclusions

Different extraction techniques can lead to varying metabolomic profiles between cell lines and mouse strains, influencing their ability to be distinguished. Our study shows that the choice of extraction method impacts the metabolome coverage, which in turn affects the discrimination of genetically different mouse strains. While KOH extraction is optimal for measurements of NADPH and polyamines and polyols, it is not suitable for detection of GSH and limits the depth of discrimination between complex metabolite extracts. Therefore, this study advocates for the pursuit of parallel extractions with KOH and MeOH for comprehensive metabolomic assessments involving redox signaling. Future studies are warranted to determine whether inhibition of NNT, which increases NADPH but reduces GSH/GSSG ratio in hepatocytes, may afford protection or predisposes to the development of HCC or SLE.

## Data Availability

The original contributions presented in this study are included in the article/Supplementary Material. Further inquiries can be directed to the corresponding author.

## Supplementary Materials

The following supporting information can be downloaded at https://www.mdpi.com/article/10.3390/ijms262110371/s1.

## Abbreviations

FA	formic acid
GSH	reduced glutathione
GSSG	oxidized glutathione
HCC	hepatocellular carcinoma
HPLC-UV	high-performance liquid chromatography with ultraviolet detection
KOH	potassium hydroxide
LC-MS	liquid chromatography combined with mass spectrometry
MeOH	methanol
NADPH	reduced form of nicotinamide adenine dinucleotide phosphate
NADP	oxidized form of nicotinamide adenine dinucleotide phosphate
NADH	reduced form of nicotinamide adenine dinucleotide
NAD	oxidized form of nicotinamide adenine dinucleotide
SLE	systemic lupus erythematosus
