# Supplementary material for: Potassium-Hydroxide-Based Extraction of Nicotinamide Adenine Dinucleotides from Biological Samples Offers Accurate Assessment of Intracellular Redox Status
Source: Int J Mol Sci. 2025 Oct 24;26(21):10371. doi: 10.3390/ijms262110371 (PMC12607542; doi:10.3390/ijms262110371)
Supplement: Supplementary file 1 [file ijms-26-10371-s001.zip › ijms-3915074-supplementary.pdf]

Figure S1 Liver Mitochondria

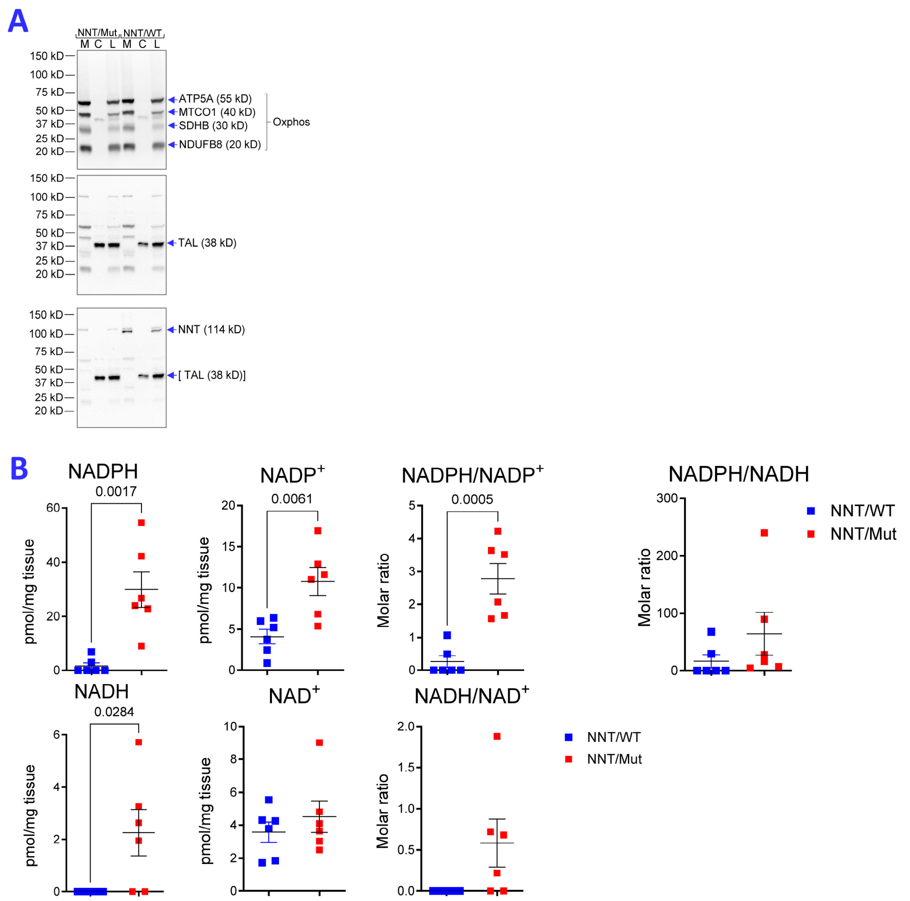

Figure S1. Effect of NNT deficiency on reduced and oxidized pyridine nucleotide content in isolated liver mitochondria. A, Western blot analysis of isolated mitochondria. Isolation of mitochondria was confirmed by Western blot detection of proteins involved in oxidative phosphorylation (Oxphos antibody coctail, Abcam Cat. No. 110413). Purity of mitochondrial fraction from contamination by cytosol was assessed by Western blot detection of cytosolic enzyme, transaldolase (TAL), using rabbit polyclonal antibody 169 (26). NNT was confined to mitochondria as detected with a rabbit polyclonal antibody (Sigma Cat. No. HPA004829). **B**, KOH extractions were performed using mitochondria isolated from the livers of six, 10-month-old, age-matched male NNT/WT and NNT/Mut mice. P values reflect analysis with one-way ANOVA with Sidak correction for multiple comparisons.

Figure S2

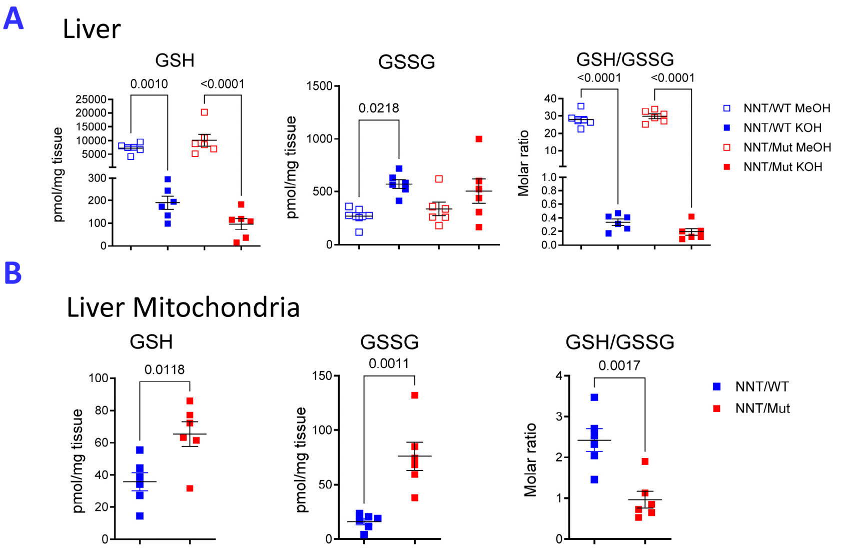

Figure S2. Effect of NNT deficiency on reduced (GSH) and oxidized glutathione (GSSG) content and GSH/GSSG ratio in the liver and isolated liver mitochondria. KOH and MeOH extractions were performed using liver tissues (**panel A**) and KOH extractions were performed from isolated liver mitochondria (**panel B**) from six, 10-month-old, age-matched male NNT/WT and NNT/Mut mice. P values reflect analysis with one-way ANOVA with Sidak correction for multiple comparisons.

Figure S3

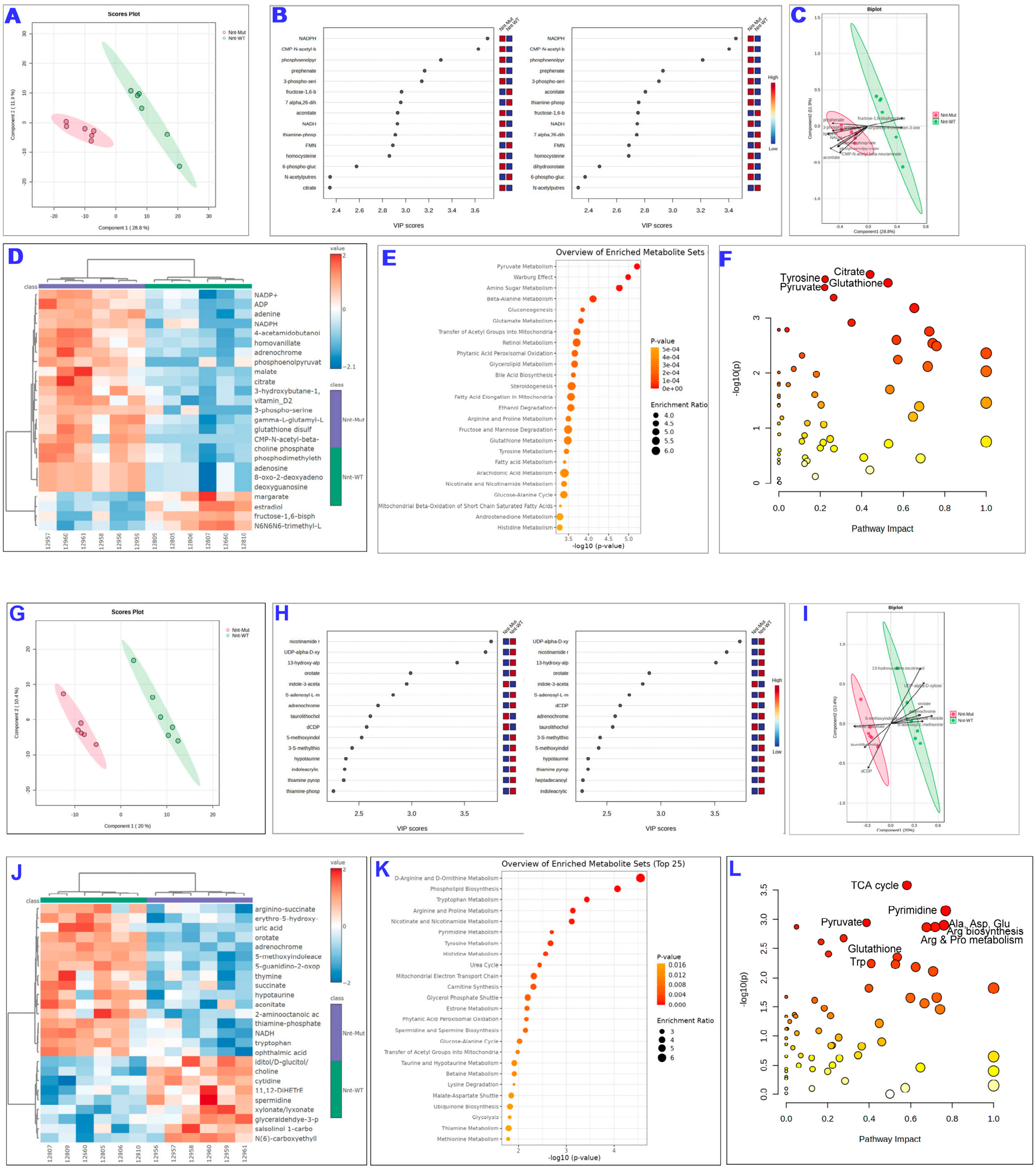

Figure S3. Compartmentalized effect of NNT deficiency on the metabolome of the liver. KOH extraction was performed using isolated mitochondria (**panels A-F**) and liver tissue (**panels G-L**) from six, 10-month-old, age-matched male NNT/WT and NNT/Mut mice. A, Discrimination of the metabolomes between mitochondria from NNT/WT and NNT/Mut mice by partial least square discriminant analysis (PLS-DA). B, Variable importance in projection (VIP) scores of top 15 metabolites in components 1 and 2 allowing for discrimination of NNT/WT and NNT/Mut mitochondria. C, Biplot display of samples as points and metabolites as vectors. D, Heat diagram of metabolites discriminating NNT/WT and NNT/Mut mitochondria. E, Enrichment diagram of biological pathways statistically impacted by observed metabolite changes. F, Two-dimensional map of affected metabolic pathways ranked by their statistical significance (p-value, Y axis) and topological pathway impact value (X axis). G, Discrimination of the metabolomes between livers of NNT/WT and NNT/Mut mice by PLS-DA. H, Variable importance in projection (VIP) scores of top 15 metabolites in components 1 and 2 allowing for discrimination of

NNT/WT and NNT/Mut livers. **I**, Biplot display of samples as points and metabolites as vectors **J**, Heat diagram of metabolites discriminating NNT/WT and NNT/Mut livers. **K**, Enrichment diagram of biological pathways statistically impacted by metabolite changes. **L**, Two-dimensional map of affected metabolic pathways ranked by their statistical significance (p-value, Y axis) and topological pathway impact value (X axis).

Figure S4

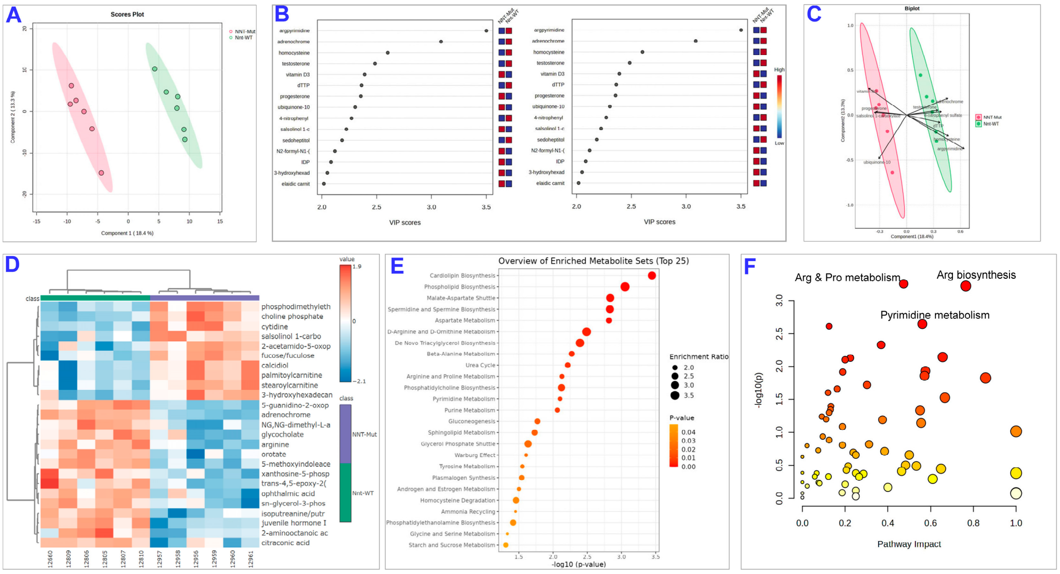

Figure S4. Discrimination of metabolomes between liver tissues of NNT/WT and NNT/Mut mice following MeOH extraction. MeOH extractions were performed from the livers of six, 10-month-old, age-matched male NNT/WT and NNT/Mut mice. **A**, Discrimination of the metabolomes between MeOH-extracted livers by PLS-DA. **B**, VIP scores of top 15 metabolites in components 1 and 2 allowing for discrimination of NNT/WT and NNT/Mut livers. **C**, Biplot display of samples as points and metabolites as vectors. **D**, Heat diagram of metabolites discriminating NNT/WT and NNT/Mut livers. **E**, Enrichment diagram of biological pathways statistically impacted by observed metabolite changes. **F**, Two-dimensional map of affected metabolic pathways ranked by their statistical significance (p-value, Y axis) and topological pathway impact value (X axis). .

Figure S5

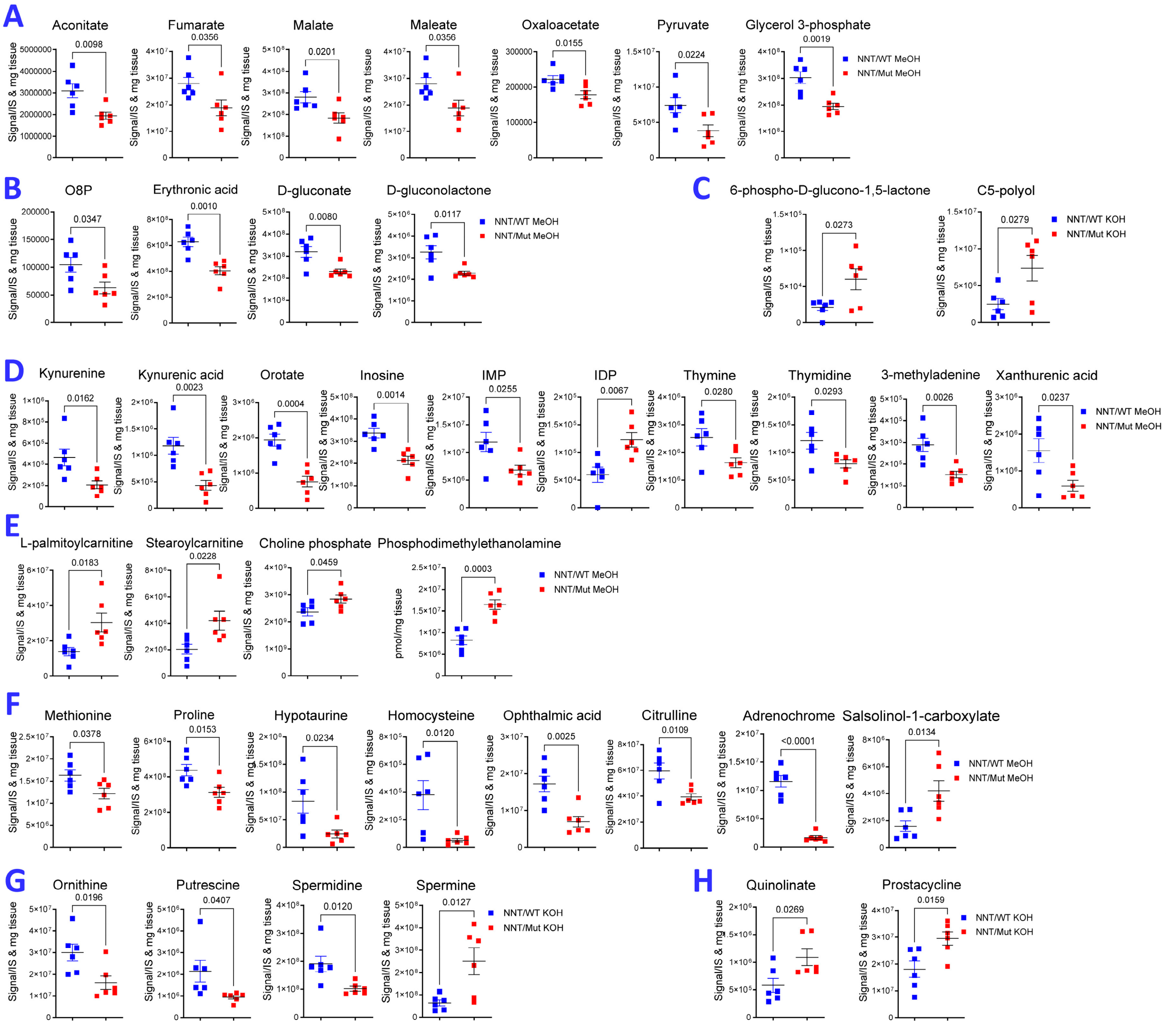

Figure S5. KOH and MeOH extractions preferentially discriminate the metabolomes between livers of NNT/WT and NNT/Mut mice. A, MeOH extraction detects depletion of TCA metabolites in NNT/Mut mice. **B**, MeOH extraction detects depletion of PPP metabolites in NNT/Mut mice. **C**, KOH extraction detects the accumulation of 6-phospho-D-glucono-1,5-lactone and C5-polyols in NNT/Mut mice. **D**, MeOH extraction detects kynurenine metabolites and nucleotides in NNT/Mut mice. **E**, MeOH extraction detects the accumulation of acylcarnitine and phospholipids in NNT/Mut mice. **F**, MeOH extraction preferentially discriminates amino acid metabolites between NNT/WT and NNT/Mut mice. **G**, KOH extraction preferentially discriminates polyamines between NNT/WT and NNT/Mut mice. **H**, KOH extraction detects quinolinate and prostacyclin accumulation in NNT/Mut mice.

Figure S6

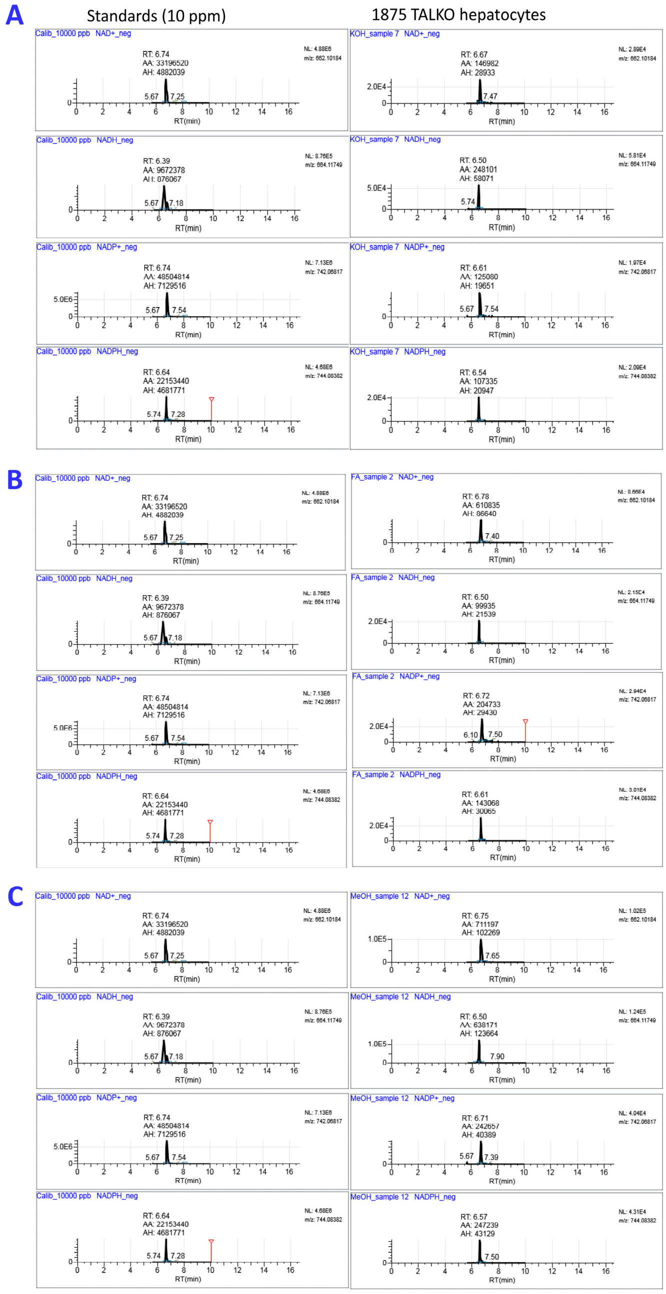

Figure S6. Detection of pyridine nucleotide standards following KOH (panel A), FA (panel B), and MeOH extraction (panel C). LC/MS measurements were performed on a Thermo Scientific Vanquish HPLC coupled to a Thermo Scientific Q Exactive hybrid quadrupole-orbitrap MS. The metabolites were separated using a hydrophilic interaction liquid-chromatography (HILIC) method on a Waters Xbridge BEH Amide column. Retention times and m/z values of NADPH, $NADP^+$, NADH, and NAD^+ are indicated following detection in negative on mode for each extraction.

Figure S7

A Detected Metabolites

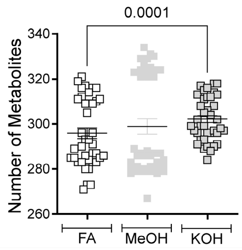

All metabolites without treatment

B Detected Metabolites

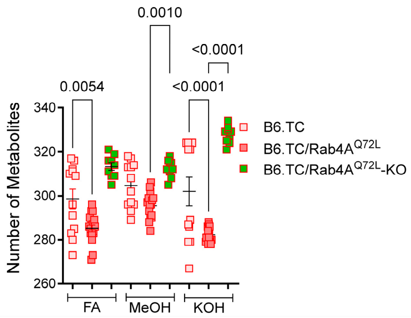

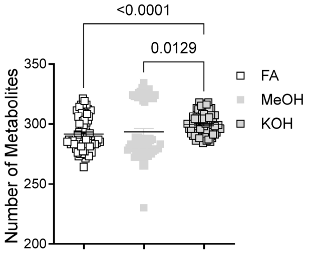

All metabolites with treatment

Figure S7. KOH extraction is superior to FA or MeOH for breadth of metabolite detection and discrimination between Rab4-induce metabolic changes in 39 female lupus-prone mice. A, Comparison of the numbers of metabolites that were detectable after KOH, FA, or MeOH extraction in >75% of samples across all genotypes. B, Comparison of the numbers of measurable metabolites between 5-month-old, age-matched female B6.TC/Rab4A^{Q72L} (n=18), B6.TC/Rab4A^{Q72L}-KO (n=9), and B6TC hepatocytes (n=12). P values reflect analysis with one-way ANOVA with Sidak correction for multiple comparisons.
